# The antioncogenic effect of Beclin-1 and FOXP3 is associated with SKP2 expression in gastric adenocarcinoma

**DOI:** 10.1097/MD.0000000000026951

**Published:** 2021-08-20

**Authors:** Hyung Kyung Kim, Kyu Yeoun Won, Sang-Ah Han

**Affiliations:** a Department of Pathology, Kyung Hee University Hospital at Gangdong, School of Medicine, Kyung Hee University, Seoul, Korea; b Department of Surgery, Kyung Hee University Hospital at Gangdong, School of Medicine, Kyung Hee University, Seoul, Korea.

**Keywords:** Beclin-1, forkhead box protein P3, gastric adenocarcinoma, regulatory T cells, S-phase kinase-associated protein 2

## Abstract

An overexpression of S-phase kinase-associated protein 2 (SKP2) is frequently observed in human cancer progression and metastasis, and evidence suggests that *SKP2* plays a proto-oncogenic role both in vitro and in vivo. However, the function of SKP2 in gastric adenocarcinoma remains largely obscure. We investigated SKP2 expression in human gastric carcinomas.

Tissue samples were acquired from 182 cases of gastric adenocarcinoma that were surgically resected from 2006 to 2012. Immunohistochemical staining for SKP2, Beclin-1, and forkhead box protein P3 (FOXP3) was performed. Pearson chi-square test was used to evaluate the associations among clinicopathological variables. The Kaplan–Meier method, the log-rank test, and the Cox proportional-hazards model were used in the analysis of the overall survival (OS) and disease-free survival (DFS).

As a result, SKP2 overexpression in gastric adenocarcinomas showed a significant correlation with several favorable clinical factors, including the tumor size, T category, N category, lymphatic invasion, vascular invasion, OS, and DFS. SKP2 expression was positively correlated with the tumoral FOXP3, Beclin-1 expression, and regulatory T cell (Treg) infiltration. The difference in DFS between the SKP2 positive and negative group was attenuated by FOXP3 high expression, Beclin-1 high expression, and Tregs infiltration. Attenuation of the difference in OS by FOXP3 high expression, Beclin-1 high expression, and Tregs infiltration was not significant. In multivariable analysis, SKP2 expression was not correlated with OS and DFS.

Our study showed a complex interrelationship between SKP2 and Beclin-1 and FOXP3 expression in gastric adenocarcinoma. The antioncogenic effect of Beclin-1 and FOXP3 expression in gastric adenocarcinoma is related to SKP2 expression.

## 1 Introduction

S-phase kinase-associated protein 2 (SKP2) is a component of the E3 ubiquitin ligase SKP1–Cul1–Fbox complex.^[1]^ SKP2 is the substrate-recruiting component of the SCF–SKP2 complex, which targets cell cycle control elements, such as p27 and p21. Here, SKP2 has been implicated in double negative feedback loops with both p21 and p27, that control the cell cycle entry and G1/S transition.^[2,3]^*SKP2* is considered a proto-oncogene as its overexpression results in a proliferation increase, at least in part, through an increased p27 proteolysis.^[4]^

SKP2 overexpression is frequently observed in human cancer progression and metastasis, and evidence suggests that *SKP2* plays a proto-oncogenic role both in vitro and in vivo.^[5]^ SKP2 overexpression has been seen in lymphomas, prostate cancer, melanoma, nasopharyngeal carcinoma, pancreatic cancer, and breast carcinomas.^[6–11]^ Additionally, the overexpression of SKP2 is correlated with a poor prognosis in breast cancer.^[12]^ However, the function of SKP2 in gastric adenocarcinoma remains largely obscure.

Forkhead box protein P3 (FOXP3) expression is observed in cancer cells and infiltrated regulatory T cells (Tregs). In recent studies, FOXP3 was suggested to play an important role in tumor development, in addition to its association with Treg function in the immune system.^[13]^ FOXP3 expression in tumor cells has been reported in pancreatic cancer, melanoma, and other tumor cell lines.^[14]^ The role of the *FOXP3* gene in tumor development and the mechanisms that regulate FOXP3 expression has increasingly generated interest from researchers. Zuo et al reported that FOXP3 is a novel transcriptional repressor for the breast cancer oncogene *SKP2*.^[1]^

In a recent study, the interrelationship between SKP2 and Beclin-1, an autophagy-related protein, was reported. The authors reported that genetic or pharmacological inhibition of SKP2 decreases Beclin-1 ubiquitination.^[15]^

In our previous study, we concluded that the expression of the autophagy-related protein, Beclin-1, was associated with tumor FOXP3 expression and Tregs in gastric adenocarcinoma and that Beclin-1 expression acts as a favorable prognostic factor in gastric adenocarcinoma.^[16]^

Therefore, this study was initiated as a part of finding out how the expression and interaction of substances that regulate tumor proliferation and the tumor microenvironment in gastric cancer affect the clinical prognosis. In the present study, the expression of SKP2 and its relationship with Beclin-1 and FOXP3 expression in gastric adenocarcinoma were investigated.

## 2 Materials and methods

### 2.1 Patients and tissue samples

Tissue samples were acquired from 182 cases of gastric adenocarcinoma that were surgically resected at Kyung Hee University Hospital at Gangdong from 2006 to 2012. Inclusion criteria were histologically confirmed gastric adenocarcinoma, staged pathologically stage I–III, at least 15 examined lymph nodes are required to ensure the adequate tumor nodes metastases classification. Patients who underwent curative D2 lymphadenectomy resection for gastric cancer with no macroscopic or microscopic evidence for remaining tumor. Patients with evidence of any distant metastatic disease were excluded. Two pathologists (K.Y. Won and H.K. Kim) reviewed all the original hematoxylin and eosin (H&E)-stained slides. The clinicopathological variables, including age, sex, recurrence, tumor type, histologic grade, tumor size, presence of lymphatic, vascular, and perineural invasion, primary tumor category (pT), primal nodal and distant metastases, were evaluated. Of the 182 patients, 31 (16.9%) died of the disease, and 133 (72.7%) were alive on the day the study was initiated. The mean age of patients was 63.5 years (range, 34–93 years). This study was approved by the Institutional Review Board of Kyung Hee University Hospital at Gangdong (IRB 2016-07-006-001).

### 2.2 Tissue microarray (TMA) construction

The H&E-stained, formalin-fixed, and paraffin-embedded tumor tissue sections were screened to identify the viable representative areas of gastric adenocarcinoma. The corresponding areas on the tissue block were then marked for tissue core punches. The tissue microarrays (TMAs) were assembled using a commercially available manual tissue microarrayer (Quick-Ray; UNITMA Co., Ltd, Seoul, Korea) as described in previous studies.^[16,17]^ Briefly, 3 representative tumor cores (with a diameter of 2.0 mm) were punched from each tumor tissue block, and each core was arrayed into 3 recipient paraffin blocks. Three cores per case were arrayed to increase the concordance rate between the immunohistochemistry results of the TMAs and the whole sections. Each TMA block also contained 4 normal gastric tissue cores. Each block was stained with H&E to verify the tumor cell content. Cases with only stromal tissue or insufficient carcinoma tissue in the core were excluded from the analysis. Slides were serially sectioned, and H&E staining was performed.

### 2.3 Immunohistochemical staining

Immunohistochemistry was performed on 4-μm tissue sections from each TMA block using the Bond Polymer Intense Detection system (Vision BioSystems, Victoria, Australia), according to the manufacturer's instructions with minor modifications as previously reported.^[16,18]^ In brief, 4-μm formalin-fixed, paraffin-embedded tissue sections were deparaffinized using a Bond Dewax Solution (Vision BioSystems), and an antigen retrieval procedure was performed using Bond ER Solution (Vision BioSystems) for 30 minutes at 100°C. Endogenous peroxidase activity was quenched by incubating the tissue with hydrogen peroxide for 5 minutes. The sections were incubated for 15 minutes at room temperature with primary polyclonal antibodies to SKP2 (1:100, Abcam, Cambridge, UK), Beclin-1 (1:100, Abcam), and FOXP3 (1:100, PCH101, eBioscience, Cambridge, UK) using a biotin-free polymeric horseradish peroxidase-linker antibody conjugate system and a Bond-max automatic slide stainer (Vision BioSystems). The nuclei were counterstained with hematoxylin. The negative control was treated in an identical manner using mouse IgG instead of primary antibody.

### 2.4 Evaluation of immunohistochemical staining

The SKP2 expression based on immunohistochemical staining appeared as nuclear staining with fine granular cytoplasmic staining. Normal gastric mucosal glands showed a strong nuclear SKP2 expression. The Beclin-1 expression based on immunohistochemical staining appeared as fine granular and diffuse cytoplasmic staining with occasional nuclear staining. Immunohistochemical staining of SKP2 and Beclin-1 was evaluated based on the staining intensity and proportion. The intensity score was defined as 0 (no staining), 1 (weak staining), 2 (moderate staining), and 3 (strong staining). The proportion score was defined as 1 (<30% of tumor cells) and 2 (≥30% of tumor cells). The intensity score and the proportion score were multiplied to obtain a total score. Total scores were as follows: 0 to 1 (negative) and 2 to 6 (positive). Tumoral FOXP3 expression was observed in the nuclei and cytoplasm of carcinoma cells. The staining of at least 20% of cells (either in the nucleus or in the cytoplasm) was considered as a positive FOXP3 expression.^[19]^ FOXP3 expression in Tregs appeared as nuclear staining. The number of FOXP3-expressing Tregs in the tumoral epithelium and stroma was counted 3 separate times under a high-power field (HPF, ×400 magnification), and the average scores were correlated with the clinicopathological variables. Cases with ≥15 FOXP3-positive cells/HPF were defined as higher density cases of infiltrated Tregs.^[20]^ All slides were evaluated independently by 2 investigators (K.Y. Won and H.K. Kim) without knowledge of the patient identity or the clinical outcome.

### 2.5 Statistical analysis

Pearson chi-square test was used to evaluate the associations among SKP2 expression, tumoral FOXP3, and Beclin-1 expression, and Tregs, as well as several clinicopathological variables. The Kaplan–Meier method was used to determine the probability of disease-free survival (DFS) and overall survival (OS), and the data were analyzed using the log-rank test. For multivariate survival analysis, the Cox regression hazard model was used. OS was defined as survival from the date of surgery to the date of death due to cancer. A *P*-value < .05 was considered statistically significant. The statistical analyses were performed using the SPSS software package (version 15.0; SPSS, Inc., Chicago, IL).

## 3 Results

### 3.1 Relationship among S-phase kinase-associated protein 2 expression, tumoral forkhead box protein P3 expression, and several clinicopathological variables

**Figure 1:**
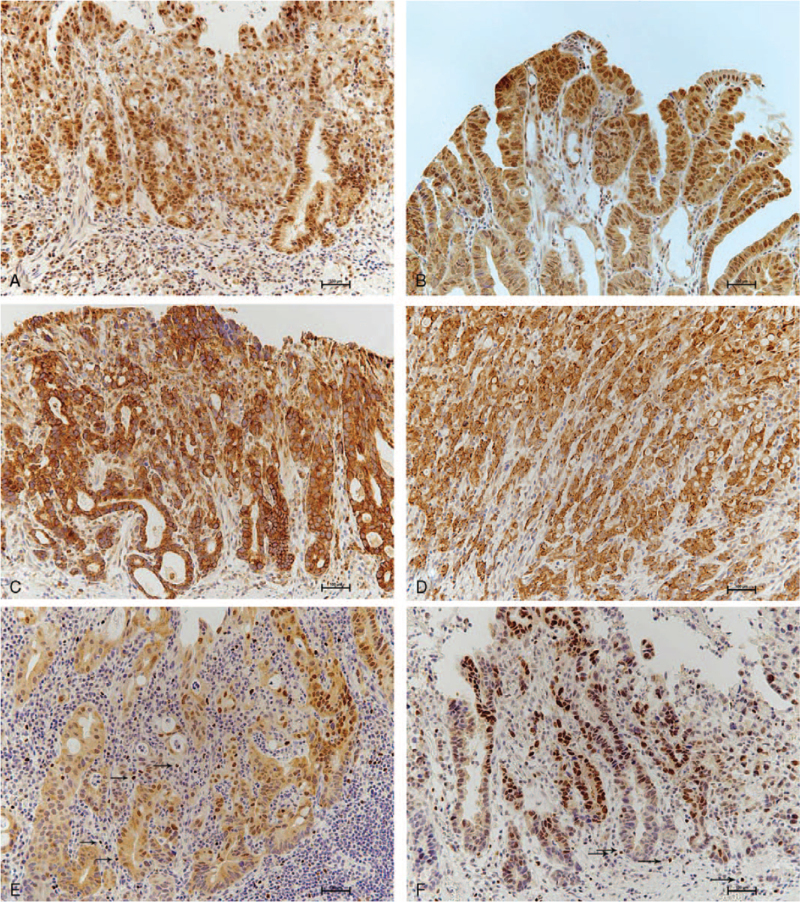
Representative photographs of SKP2, Beclin-1, tumoral FOXP3 expression and infiltrated Tregs in the stroma in gastric adenocarcinoma (A–F). A. A positive SKP2 expression is both present in the nucleus and cytoplasm in poorly differentiated adenocarcinomas. B. Well-differentiated adenocarcinomas show a strong nuclear and granular cytoplasmic SKP2 expression. C. Positive cytoplasmic Beclin-1 expression is found in a moderately differentiated adenocarcinoma. D. Poorly differentiated adenocarcinoma cells show a strong cytoplasmic Beclin-1 expression. E. Positive tumoral FOXP3 expression is observed in both the nuclei and cytoplasm of gastric adenocarcinoma cells. Scattered FOXP3-positive lymphoid cells (Tregs) (black arrow) are identified in the tumor stroma. F. Another case of gastric adenocarcinoma also shows both nuclear and cytoplasmic FOXP3 expression in gastric adenocarcinoma cells. Scattered FOXP3-positive lymphoid cells (Tregs) (black arrow) are identified in the tumor stroma. FOXP3 = forkhead box protein P3, SKP2 = S-phase kinase-associated protein 2, Tregs = regulatory T cells.

A positive SKP2 expression was observed in 47.8% (87/182) of the gastric adenocarcinoma samples (Fig. 1A, B). A positive Beclin-1 expression was observed in 59.3% (108/182) of the gastric adenocarcinoma samples (Fig. 1C, D). A positive tumoral FOXP3 expression was observed in 49.2% (90/182) of the gastric adenocarcinoma cases. Infiltration of FOXP3-expressing Tregs (≥15 FOXP3-positive cells/HPF) was observed in 40.7% (74/182) of cases (Fig. 1E, F). The normal gastric mucosal epithelial cells were negative for FOXP3 expression.

As shown in Table 1, a positive SKP2 expression was significantly associated with smaller tumor size, lower T category, lower N category, lower stage group, lower recurrence rate, less lymphatic invasion, and less vascular invasion. A positive tumoral FOXP3 expression was significantly associated with a lower T category, lower N category, lower stage group, lower recurrence rate, and less lymphatic invasion.

**Table 1:** Correlation between SKP2 and tumoral FOXP3 expression and clinicopathological variables in 182 gastric adenocarcinomas.

		SKP2 expression			Tumoral FOXP3 expression		
	N	Negative	Positive	*P* value	Negative	Positive	*P* value
Tumor size							
≤3 cm	63	20 (31.7)	43 (68.3)	<.0001^∗^	27 (42.9)	36 (57.1)	.088
>3 cm	119	75 (63.0)	44 (37.0)		65 (54.6)	54 (45.4)	
Histologic type							
Mixed	22	11 (50.0)	11 (50.0)	.502	15 (68.2)	7 (31.8)	.061
Tubular	160	84 (52.5)	76 (47.5)		77 (48.1)	83 (51.9)	
Histologic grade							
Well/moderately	93	48 (51.6)	45 (48.4)	.524	43 (46.2)	50 (53.8)	.166
Poorly	88	46 (52.3)	42 (47.7)		48 (54.5)	40 (45.5)	
Primary tumor (T)							
I/II	101	34 (33.7)	67 (66.3)	<.0001^∗^	45 (44.6)	56 (55.4)	.049^∗^
III/IV	81	61 (75.3)	20 (24.7)		47 (58.0)	34 (42.0)	
Lymph node metastasis (N)							
Absent	100	41 (41.0)	59 (59.0)	.001^∗^	41 (41.0)	59 (59.0)	.003^∗^
Present	82	54 (65.9)	28 (34.1)		51 (62.2)	31 (37.8)	
Stage							
I to IIA	111	43 (38.7)	68 (61.3)	<.0001^∗^	46 (41.4)	65 (58.6)	.002^∗^
IIB to IIIB	71	52 (73.2)	19 (26.8)		46 (64.8)	25 (35.2)	
Recurrence							
Absent	138	61 (44.2)	77 (55.8)	<.0001^∗^	60 (43.5)	78 (56.5)	<.0001^∗^
Present	35	29 (82.9)	6 (17.1)		27 (77.1)	8 (22.9)	
Lymphatic invasion							
Absent	90	36 (40.0)	54 (60.0)	.001^∗^	37 (41.1)	53 (58.9)	.009^∗^
Present	92	59 (64.1)	33 (35.9)		55 (59.8)	37 (40.2)	
Vascular invasion							
Absent	170	85 (50.0)	85 (50.0)	.024^∗^	86 (50.6)	84 (49.4)	.601
Present	12	10 (83.3)	2 (16.7)		6 (50.0)	6 (50.0)	
Neural invasion							
Absent	161	81 (50.3)	80 (49.7)	.119	78 (48.4)	83 (51.6)	.090
Present	21	14 (66.7)	7 (33.3)		14 (66.7)	7 (33.3)	

### 3.2 Interrelationship among S-phase kinase-associated protein 2 expression, Beclin-1 expression, tumoral forkhead box protein P3 expression, and regulatory T cells in gastric adenocarcinoma

SKP2 expression was positively correlated with the tumoral FOXP3 overexpression (*P* < .0001), Beclin-1 expression (*P* < .0001), and Treg infiltration (*P* = .031) in gastric adenocarcinoma (Table 2).

**Table 2:** Correlation among SKP2, tumoral FOXP3, Beclin-1, and Tregs in 182 gastric adenocarcinomas.

	Tumoral FOXP3 expression			Beclin-1 expression			Tregs		
	Negative	Positive	*P* value	Negative	Positive	*P* value	<15/HPFs	≥15/HPFs	*P* value
SKP2 expression			<.0001^∗^			<.0001^∗^			.031^∗^
Negative	61 (64.2)	34 (35.8)		55 (57.9)	40 (42.1)		62 (66.0)	32 (34.0)	
Positive	31 (35.6)	56 (64.4)		18 (20.9)	68 (79.1)		44 (51.2)	42 (48.8)	

### 3.3 Relationship between S-phase kinase-associated protein 2 expression, Beclin-1 expression, tumoral forkhead box protein P3 expression, regulatory T cells, and survival analysis

**Figure 2:**
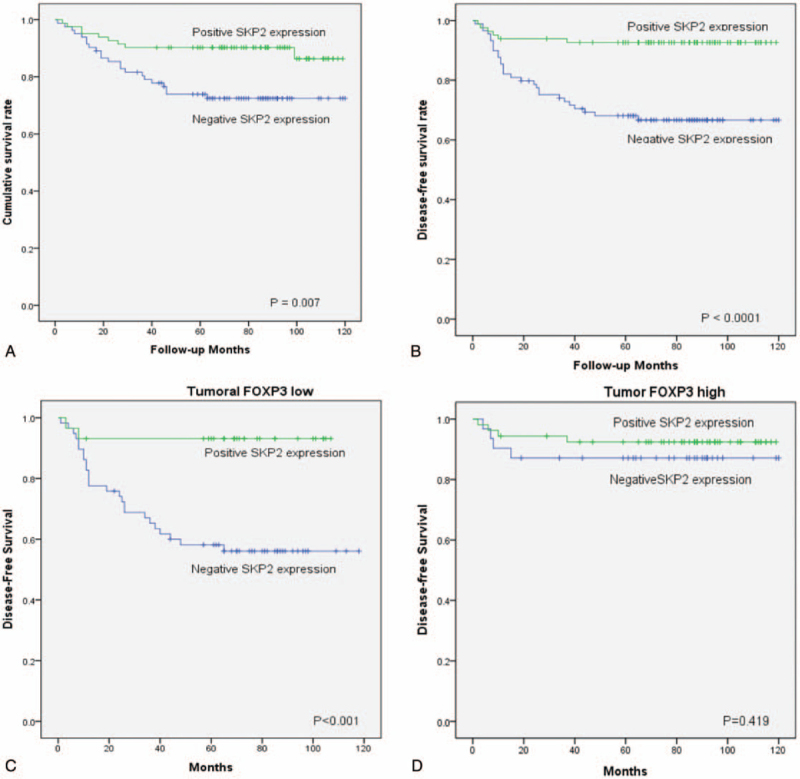
Analysis of the overall survival and disease-free survival rates based on SKP2 expression in gastric adenocarcinoma. A. The patients with a positive SKP2 expression showed a significantly higher cumulative survival rate than patients with a negative SKP2 expression in gastric adenocarcinoma (*P* = .007). B. The patients with a positive SKP2 expression showed a significantly higher disease-free survival rate than the patients with a negative SKP2 expression (*P* < .0001). C. The patients with a positive SKP2 expression presented a significantly higher disease-free survival rate than the patients with a negative SKP2 expression among the tumoral FOXP3 population (*P* < .001). D. Difference in disease-free survival between positive SKP2 expression and negative SKP2 expression is attenuated among tumoral FOXP3 population (*P* = .419). FOXP3 = forkhead box protein P3, SKP2 = S-phase kinase-associated protein 2.

Adequate clinical follow-up information was available for each of the 182 patients with gastric adenocarcinoma. As shown in Table 3, the univariate analyses for OS revealed an association with SKP2 expression (*P* = .007) (Fig. 2A), Beclin-1 expression (*P* < .0001), tumoral FOXP3 expression (*P* = .009), the number of Tregs (*P* = .012), a larger tumor size (*P* = .016), pT (*P* < .0001), lymph node metastasis (*P* = .0003), lymphatic invasion (*P* = .004), and recurrence (*P* < .0001). The univariate analyses for DFS revealed an association with SKP2 expression (*P* < .0001) (Fig. 2B), Beclin-1 expression (*P* < .0001), tumoral FOXP3 expression (*P* = .001), the number of Tregs (*P* < .0001), a larger tumor size (*P* = .001), pT (*P* < .0001), lymph node metastasis (*P* < .0001), lymphatic invasion (*P* < .0001), and neural invasion (*P* = .0367).

**Table 3:** Univariate analysis of clinicopathological variables for overall survival rate in 182 gastric adenocarcinomas.

Variables	Disease-free survival (*P* value)	Overall survival (*P* value)
Tumor size (<3.0 cm vs ≥3.0 cm)	.0010^∗^	.0163^∗^
Tumor type (tubular vs mixed)	.2419	.3431
Histologic grade (well to mod vs poor)	.0380^∗^	.1868
Primary tumor (T) (I, II vs III, IV)	<.0001^∗^	<.0001^∗^
Lymph node metastasis	<.0001^∗^	.0003^∗^
Recurrence	N.A	<.0001^∗^
Lymphatic invasion	.0041^∗^	.0040^∗^
Vascular invasion	.6434	.6211
Neural invasion	.0367^∗^	.0953
Tumoral FOXP3 expression	.001^∗^	.009^∗^
Tregs count (<15/HPFs, ≥15/HPFs)	<.0001^∗^	.012^∗^
Beclin-1 expression	<.0001^∗^	<.0001^∗^
SKP2 expression	<.0001^∗^	.007^∗^

OS and DFS time are presented in comparison with the positive SKP2 group and negative SKP2 group in Table 4. It is stratified according to the expression of tumoral FOXP3 expression, Beclin-1 expression, and Tregs infiltration. Good OS with positive SKP2 expression was maintained with low tumoral FOXP3 (*P* = .050) and Tregs infiltration (*P* = .020). However, the OS difference between positive and negative SKP2 expression was attenuated with high tumoral FOXP3 expression and high Tregs infiltration. Better DFS with positive SKP2 expression was maintained with low tumoral FOXP3 (*P* = .001, Fig. 2C), low Beclin-1 (*P* = .037). With high tumoral FOXP3 (Fig. 2D) and high Beclin-1, DFS difference was not significant. Positive SKP2 expression was correlated with better DFS regardless of magnitudes in Tregs infiltration.

**Table 4:** Overall survival and disease free survival between SKP2 expression and survival difference attenuation by overexpression of tumoral FOXP3 expression, Beclin-1 expression and Tregs infiltration.

	Positive SKP2 expression		Negative SKP2 expression		
Overall survival	Mean (months)	95% CI	Mean (months)	95% CI	*P*
Tumoral FOXP3 expression					
High	110.2	102.8 to 117.6	105.9	93.0 to 118.8	.475
Low	94.2	82.5 to 105.9	85.7	73.5 to 97.9	.050
Beclin-1 expression					
High	108.3	101.6 to 114.9	109.2	99.0 to 119.3	.733
Low	98.8	78.1 to 119.5	79.7	65.7 to 93.8	.153
Tregs count					
≥15/HPFs	107.7	99.6 to 115.7	106.9	94.8 to 118.9	.423
<15/HPFs	105.0	94.4 to 115.6	85.6	73.0 to 98.2	.020^∗^

	Positive SKP2 expression		Negative SKP2 expression		
Disease-free survival	Mean (months)	95% CI	Mean (months)	95% CI	*P*
Tumoral FOXP3 expression					
High	110.9	103.4 to 118.6	105.6	92.4 to 118.8	.419
Low	100.0	90.6 to 109.4	75.8	63.2 to 88.5	.001^∗^
Beclin-1 expression					
High	110.7	104.6 to 116.7	104.3	92.7 to 115.9	.123
Low	103.9	84.3 to 123.4	71.8	57.6 to 86.1	.037^∗^
Tregs count					
≥15/HPFs	N.A	N.A	N.A	N.A	.010^∗^
<15/HPFs	N.A	N.A	N.A	N.A	.008^∗^

### 3.4 Multivariable analysis of overall survival and disease-free survival

Multivariable survival analysis was performed. Included factors were primary tumor (T) (I, II vs III, IV), N stage (N0 vs N1–3), tumor grade (well to mod vs poor), lymphatic invasion, vascular invasion, neural invasion, SKP2 expression, Beclin-1 expression, tumoral FOXP3 expression, and number of Tregs (<15/HPFs, ≥15/HPFs).

Significant factors for OS was primary tumor (T) stage [OR 3.017, 95% Confidence interval (CI): 1.082–8.415, *P* = .035] and lymph node metastasis [OR 2.977, 95% CI: 1.065–8.320, *P* = .038] Significant poor prognostic factors for DFS was primary tumor (T) stage [OR 4.220, 95% CI: 1.382–12.887, *P* = .011] and lymph node metastasis [OR 3.857, 95% CI: 1.261–11.795, *P* = .018]. The more Tregs counts (≥15/HPFs) was correlated to better DFS [OR 0.264, 95% CI: 0.102–0.685, *P* = .006]. Hazard rate for DFS is grater in the Tregs counts <15/HPFs. SKP2 expression, Beclin-1 expression, and tumoral FOXP3 expression were not significant in multivariate survival analysis (OS and DFS).

## 4 Discussion

Various studies have discovered that SKP2 plays an important role in tumorigenesis in a variety of cancer types in humans.^[21,22]^ However, the function of SKP2 in gastric adenocarcinoma remains largely obscure. The Beclin-1 and FOXP3 expression in gastric adenocarcinoma were investigated in our previous study.^[16]^ As an extension of this research, we investigated the SKP2, Beclin-1, and FOXP3 expression in gastric adenocarcinoma.

Our present results about SKP2 expression in gastric adenocarcinoma showed some different aspects compared with the results of previous studies. In our study, the SKP2 expression in gastric adenocarcinomas showed a significant correlation with favorable clinical factors, including the tumor size, T category, N category, stage group, recurrence rate, lymphatic invasion, vascular invasion, OS, and DFS. However, SKP2 expression was not a significant factors for OS nor DFS in multivariable analysis. Therefore, we investigated why these results regarding SKP2 expression in gastric adenocarcinoma were obtained. In our previous study, we observed that Beclin-1 expression in gastric adenocarcinoma is associated with the regulation of Tregs and the tumoral FOXP3 expression.^[16]^ Interestingly, in the present study, we discovered that SKP2 expression was significantly related to Beclin-1 and tumoral FOXP3 expression and infiltrated Tregs in gastric adenocarcinoma. Especially, SKP2 expression was positively correlated with Beclin-1 expression in gastric adenocarcinoma. Beclin-1 is one of the key regulators in autophagy. A recent study showed that SKP2 activity is regulated by the phosphorylation of Beclin-1 and that the inhibitors of SKP2 enhance autophagy.^[15]^ Our new finding is that the SKP2 expression is closely related to Beclin-1 expression in gastric adenocarcinoma.

In contrast, the overexpression of SKP2 is frequently observed in human cancer progression and metastasis, and evidence suggests that *SKP2* plays a proto-oncogenic role both in vitro and in vivo.^[5]^ SKP2 overexpression promoted growth and tumorigenesis in a xenograft tumor model.^[23]^ Moreover, SKP2 inactivation profoundly restricted cancer development by triggering a massive cellular senescence or apoptosis response that was surprisingly observed only in oncogenic conditions in vivo.^[24]^ Taken together, we observed that SKP2 expression was positively correlated with Beclin-1 expression in gastric adenocarcinoma. Thus, the expression of SKP2 might stimulate Beclin-1 expression to suppress SKP2 activity. This finding is the first report about the relationship between SKP2 and Beclin-1 expression in gastric adenocarcinoma.

In addition, SKP2 expression was correlated with the tumoral FOXP3 expression in gastric adenocarcinoma. FOXP3 is known to be a transcriptional repressor of the oncogene *SKP2*. Zuo et al demonstrated that FOXP3-mediated repression of SKP2 occurred in normal and malignant breast epithelial cells in humans.^[1]^ However, the relationship between SKP2 and FOXP3 in gastric adenocarcinoma has not been studied before. We found that the more the cancer cells expressed SKP2, the more tumoral FOXP3 was significantly expressed. Based on these findings, we can infer that the increased oncogenic properties of SKP2 in gastric adenocarcinoma are repressed by an increased FOXP3 expression.

Moreover, we also observed that SKP2 expression was significantly correlated with Tregs. Wang et al have recently demonstrated that SKP2 is a dynamic key regulator that acts as an important functional switch between autoreactive pathogenic T cells and Tregs.^[25]^ Our study showed that SKP2 expression in cancer cells is significantly associated with infiltrated FOXP3 + Tregs in gastric adenocarcinoma. Usually, the higher the number of infiltrated FOXP3 + Tregs in cancer, the poorer the prognosis because Tregs maintain immune homeostasis and inhibit immune responses in various diseases, including cancer. However, in this study, the patients with an increased number of infiltrated Tregs (≥15/HPF) showed better DFS and OS rates.

According to a recent study, the results regarding the effects of infiltrated Tregs on prognosis are conflicting. A high density of tumor-infiltrating FOXP3 + Tregs has been associated with a poor outcome in various solid tumors, including ovarian, pancreatic, liver, and breast cancers.^[26–29]^ Conversely, in only a few studies, including ours, a favorable prognosis was associated with an increased number of infiltrated Tregs in colorectal and gastric cancers.^[30,31]^ However, the underlying mechanism is not yet clear.

Among the unknown effects of infiltrated Tregs on the prognosis of cancers, we have discovered that SKP2 expression is significantly related to Tregs in the tumor stroma of gastric adenocarcinoma. The adenocarcinomas that are heavily infiltrated with Tregs show a higher expression of SKP2. Obviously, the tumorigenic nature of SKP2 is associated with infiltrated FOXP3 + Tregs.

Our study showed a complex interrelationship among SKP2, Beclin-1, and FOXP3 expression (in the tumor cells and Tregs) in gastric adenocarcinoma. The results of our study showed that the oncogenic ability of cell proliferation and cancer promotion, represented by SKP2 expression in gastric cancer tissues, was repressed by the expression of Beclin-1 and FOXP3 and thus responded to the survival outcome. Based on the results of our study, it is reasonable to view SKP2 expression as an indicator of tumor proliferation rather than as a single prognostic factor. Clinical prognosis is not directly related to SKP2 expression, but corresponding to tumoral FOXP3, Beclin-1, and Tregs. Furthermore, in our study, it can be considered that the stage in which these molecules are involved in tumorigenesis of gastric cancer is relatively early in cancer development, given that it is related to early cancer with small tumor size or no lymph node metastasis. Similarly, expression of common gastric cancer stem cell markers, Lgr5, and DCLK1, was more expressed in early gastric cancer than in advanced gastric cancer, and these stem cell markers involved in cancer development and progression are involved in the early stage of gastric cancer development. It is predicted that a follow-up study on the microenvironment of cancer development in which SKP2, Beclin-1, tumoral FOXP3, and stem cell markers are complexly involved in the occurrence of early gastric cancer is necessary.^[17]^ It is thought to be the totality of the tumor microenvironment to which infiltration is involved. These findings suggest the possibility of understanding the mechanisms of the tumor microenvironment in gastric cancer and devising appropriate targeted therapies. In patients who experience gastric cancer progression or distant metastasis, the prognosis is still lacking. Nonetheless, the combination of ramucirumab, a recombinant human immunoglobulin G1 monoclonal antibody that binds to the extracellular binding domain of VEGFR-2, with paclitaxel is widely considered the optimal second line of treatment in metastatic GC.^[32,33]^ The study result of gastric cancer indicate a lower efficacy of second-line paclitaxel + ramucirumab in the treatment of advanced GC in patients with lung metastases. Recently, several drugs have been shown to increase survival against placebo in advanced GC; apatinib, a small selective VEGFR2 tyrosine kinase inhibitor, is now approved in China,^[34]^ nivolumab, a monoclonal antibody inhibitor of programmed death-1, is approved in Japan^[35,36]^ and, more recently, trifluridine/tipiracil, a novel oral combination cytotoxic drug, also known as TAS-102.^[37]^ Although these treatments are effective, the absolute survival benefit is only 2 to 3 months compared to the control group. Due to these practical limitations, it is considered that efforts to discover other therapeutic targets in gastric cancer should be continued. From this point of view, understanding the cancer microenvironment through our research is valuable in that it shows the possibility of discovering new therapeutic targets.

In conclusion, the antioncogenic effect of Beclin-1 and FOXP3 expression in gastric adenocarcinoma is corresponding to SKP2 expression. Moreover, the action of Tregs in tumor stroma is influenced by the tumor SKP2 expression in gastric adenocarcinoma.

## Author contributions

**Conceptualization:** Kyu Yeoun Won.

**Data curation:** Hyung Kyung Kim.

**Formal analysis:** Hyung Kyung Kim, Kyu Yeoun Won, Sang-Ah Han.

**Investigation:** Hyung Kyung Kim, Kyu Yeoun Won.

**Methodology:** Hyung Kyung Kim, Kyu Yeoun Won.

**Supervision:** Kyu Yeoun Won, Sang-Ah Han.

**Validation:** Hyung Kyung Kim, Kyu Yeoun Won.

**Visualization:** Hyung Kyung Kim.

**Writing – original draft:** Hyung Kyung Kim.

**Writing – review & editing:** Kyu Yeoun Won, Sang-Ah Han.
