# The antioncogenic effect of Beclin-1 and FOXP3 is associated with SKP2 expression in gastric adenocarcinoma: Erratum

**DOI:** 10.1097/MD.0000000000027193

**Published:** 2021-09-10

In the article, “The antioncogenic effect of Beclin-1 and FOXP3 is associated with SKP2 expression in gastric adenocarcinoma”,^[1]^ which appears in Volume 100, Issue 33 of *Medicine*, Figure 2B was incorrect and has been corrected. The accurate figure is below:

**Figure 2:**
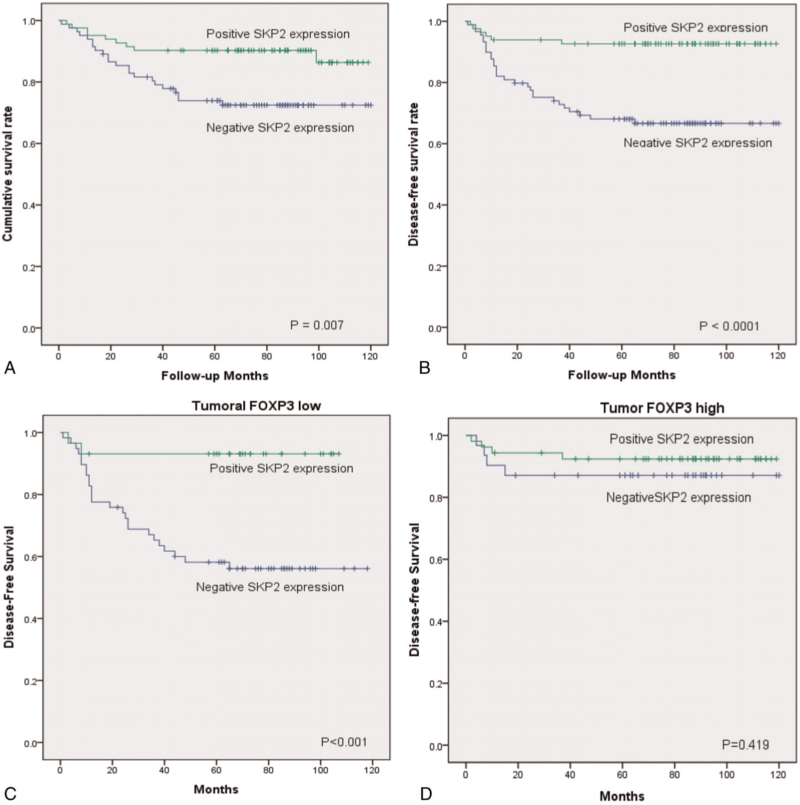
Analysis of the overall survival and disease-free survival rates based on SKP2 expression in gastric adenocarcinoma. A. The patients with a positive SKP2 expression showed a significantly higher cumulative survival rate than patients with a negative SKP2 expression in gastric adenocarcinoma (*P = *.007). B The patients with a positive SKP2 expression showed a significantly higher disease-free survival rate than the patients with a negative SKP2 expression (*P < *.0001) C. The patients with a positive SKP2 expression presented a significantly higher disease-free survial rate than the patients with a negative SKP2 expression among the tumoral FOXP3 population (*P* <.001). D. Difference in disease-free survival between positive SKP2 expression and negative SKP2 expression is attenuated among tumoral FOXP3 population (*P = *.419). FOXP3 = forkhead box protein P3, SKP2 = S-phase kinase-associated protein 2.
